# RGD-Hydrogel Improves the Therapeutic Effect of Bone Marrow-Derived Mesenchymal Stem Cells on Phosgene-Induced Acute Lung Injury in Rats

**DOI:** 10.1155/2022/2743878

**Published:** 2022-05-17

**Authors:** Jianwen Ding, Yu Dun, Daikun He, Yiru Shao, Fuli Liu, Lin Zhang, Jie Shen

**Affiliations:** ^1^Center of Emergency and Critical Medicine, Jinshan Hospital of Fudan University, 201508 Shanghai, China; ^2^Research Center for Chemical Injury, Emergency and Critical Medicine of Fudan University, 201508 Shanghai, China; ^3^Key Laboratory of Chemical Injury, Emergency and Critical Medicine of Shanghai Municipal Health Commission, 201508 Shanghai, China

## Abstract

Mesenchymal stem cells (MSCs) have promising potential in the treatment of various diseases, such as the therapeutic effect of bone marrow-derived MSCs for phosgene-induced acute lung injury (P-ALI). However, MSC-related therapeutics are limited due to poor cell survival, requiring appropriate MSC delivery systems to maximise therapeutic capacity. Biomaterial RGD-hydrogel is a potential cell delivery vehicle as it can mimic the natural extracellular matrix and provide cell adhesion support. The application of RGD-hydrogel in the MSC treatment of respiratory diseases is scarce. This study reports that RGD-hydrogel has good biocompatibility and can increase the secretion of Angiopoietin-1, hepatocyte growth factor, epidermal growth factor, vascular endothelial cell growth factor, and interleukin-10 *in vitro* MSCs. The hydrogel-encapsulated MSCs could further alleviate P-ALI and show better cell survival *in vivo*. Overall, RGD-hydrogel could improve the MSC treatment of P-ALI by modulating cell survival and reparative activities. It is exciting to see more and more ways to unlock the therapeutic potential of MSCs.

## 1. Introduction

Phosgene is an indispensable chemical intermediate widely used in the industrial manufacturing of dyestuffs, insecticides, and pharmaceuticals. It was first used in World War I as a chemical weapon, causing thousands of casualties [1]. As a colorless, sweet smelling, and poisonous gas, it reacts with water to form hydrochloric acid and carbon dioxide [2]. Accidental exposure to large amounts of phosgene could induce acute lung injury (ALI) and even acute respiratory distress syndrome, which is characterized by endothelial and epithelial cell damage, pulmonary edema, and hemolysis [3]. Lung-protective ventilation and conservative fluid strategies are available; however, only a few effective treatments that can reverse phosgene-induced pathophysiological damage exist [4]. Therefore, it is necessary to discover novel therapeutic strategies to manage phosgene-induced ALI (P-ALI).

Mesenchymal stem cells (MSCs) are found not only in the bone marrow but also in various tissues and organs. MSCs could exert protective effects in ALI via paracrine mechanisms and cell–cell interactions, which include endothelial and epithelial permeability regulation, inflammation reduction, and tissue repair enhancement [5, 6]. Our previous studies have shown that intratracheal or intravenous infusion of bone marrow-derived MSCs could alleviate P-ALI by repairing the pulmonary air–blood barrier and inhibiting the inflammatory responses [7, 8]. However, the therapeutic effect of MSC transplantation remains limited, owing to the short survival time, low colonisation, and differentiation rate *in vivo* [9]. Moreover, most MSCs can hardly survive for 24 hours after transplantation [10]. Additionally, the local microenvironment, which is characterized by inflammatory factors and reactive oxygen species, can impair cell viability, proliferation ability, and extracellular matrix (ECM) biosynthesis ability [11]. Several strategies have been used to enhance the survival of transplanted MSCs, including preconditioning with growth factor and overexpression via genetic modification [12, 13]. However, these strategies fail to improve the adverse microenvironment that interferes with the adhesion and integration of exogenous MSCs into the host lung tissue epithelium and stroma. Biomaterials are ideal candidates for improving MSC survival and delivery in the hostile microenvironment *in vivo* owing to their good biocompatibility and biodegradability [14].

The biomaterial-hydrogel protects MSCs from the inflammatory immune system and allows biomolecule exchange, such as oxygen, cytokines, and growth factors, thus providing a suitable microenvironment for the better survival of transplanted MSCs [15]. Hydrogel modified with integrin-specific peptide RGD promotes cell–cell and cell–ECM adhesions and influences the survival and function of biomaterial delivery cells via integrin-mediated signalling [16]. Studies have reported an enhancement in the paracrine immunomodulatory ability of MSCs cultivated in RGD-hydrogel compared to the two-dimensional cultures on tissue culture plastic surfaces [17]. MSCs encapsulated in RGD-hydrogel could expedite wound healing via enhancing angiogenesis and suppressing local proinflammatory cytokines [18]. Furthermore, a subacute injection of RGD-hydrogel combined with MSCs could further promote axonal ingrowth into the lesion of spinal cord injury [19]. For the treatment of respiratory diseases, the intratracheal administration of hydrogel combined with fibroblast growth factor and interleukin-10 (IL-10) could alleviate monocutarine-induced pulmonary hypertension and bleomycin-induced pulmonary fibrosis, respectively [20, 21]. Therefore, RGD-hydrogel combined with MSCs was hypothesised to effectively improve the efficacy of P-ALI via intratracheal administration.

## 2. Materials and Methods

### 2.1. Cell Culture

Rat bone marrow-derived MSCs were purchased from Kalang Technology Co., Ltd. Shanghai, China. The MSCs were cultured in L-DMEM (Thermo Fisher Scientific, Waltham, MA, USA) and supplemented with 20% FBS (Thermo Fisher Scientific) and 100 U/ml penicillin/streptomycin (Thermo Fisher Scientific). The cultures were maintained in an incubator with 5% CO_2_ at 37°C. The MSCs were passed at 80–90% confluence in a ratio of 1 : 3, and the cells were used for subsequent experiments three generations later.

### 2.2. Microstructure of RGD-Hydrogel

The VitroGel RGD-hydrogel (The Well Bioscience Inc. NJ, USA) was lyophilized under vacuum and cut into small pieces with a thickness of 1 mm. Then, the sample was coated with gold and analyzed by a scanning electron microscope (SEM) at an accelerating voltage of 5 kV (JSM-IT200, JEOL, Japan).

### 2.3. Cytotoxicity of Different RGD-Hydrogel Concentrations

Cell Count Kit-8 (CCK-8, Beyotime, Shanghai, China) and lactate dehydrogenase (LDH) assay kit (Beyotime) were employed to assess and select RGD-hydrogel cytotoxicity and optimal concentration, respectively. Briefly, VitroGel dilution solution (The Well Bioscience Inc.) was added to VitroGel RGD-hydrogel at ratios of 1 : 0, 1 : 1, 1 : 2, 1 : 3, and 1 : 4 (RGD-hydrogel: dilution solution, v/v). The MSCs at a density of 1 × 10^6^/mL were mixed with different concentrations of RGD-hydrogel at a 1 : 4 ratio. Before determination, the cells were cultured for 1, 3, and 7 days. At each time point, the CCK-8 and LDH assays were used to detect the cell viability of MSCs and the cytotoxicity of RGD-hydrogel in MSCs, following manufacturer's protocols.

### 2.4. Biocompatibility and Degradability of RGD-Hydrogel

Nine male Sprague Dawley (SD) rats weighing 180 ± 20 g were purchased from Shanghai Jiesijie Experimental Animal Co., Ltd. (Shanghai, China) and were given free access to food and water during the experiments. After acclimatisation for 3 days, all rats were subcutaneously injected with 50 *μ*L RGD-hydrogel at each of the two sites on the back. After day 1, 3, and 7 of injection, the rats were sacrificed, and the skin tissues of the injection site (*n* = 6) were collected for histological observation using haematoxylin-eosin (HE) staining.

### 2.5. Determination of MSC-Secreted Cytokines after Culturing with RGD-Hydrogel

The MSCs were cultured in the RGD-hydrogel at different concentrations (1 : 0, 1 : 1, 1 : 2, 1 : 3, and 1 : 4 v/v). After culturing for 24 h and 48 h, the supernatant was harvested to determine the levels of cytokines Angiopoietin-1 (Ang-1), hepatocyte growth factor (HGF), epidermal growth factor (EGF), vascular endothelial cell growth factor (VEGF), and IL-10 using corresponding ELISA kits (Beyotime) following manufacturer's recommendations.

### 2.6. Establishment of P-ALI and Treatment

A total of 90 male SD rats (6-week-old, 180 ± 20 g) were purchased from Shanghai Jiesijie Experimental Animal Co., Ltd. All the rats were housed at 25 ± 2°C with 12 h light/dark cycles. After acclimatisation for 7 days, the rats were randomly divided into five groups (*n* = 18): Sham (air/PBS), PBS (P-ALI/PBS), RGD (P-ALI/RGD-hydrogel), MSC (P-ALI/MSCs), and RGD-MSC (P-ALI/RGD-hydrogel + MSCs) groups, which were used to establish a P-ALI rat model as described previously [7]. Briefly, the P-ALI rats were exposed to 8.33 mg/L phosgene for 10 min, while those in Sham group were exposed to normal indoor air. Subsequently, the rats in the MSC and RGD-MSC groups were, respectively, intratracheally instilled with MSCs and RGD-hydrogel-coated MSCs (1 × 10^6^ cells/rat). Similarly, the rats in the Sham, PBS, and RGD groups were administrated with the same amount of PBS, PBS, and RGD-hydrogel using the same method. After 6, 24, and 48 h of treatment, all rats were sacrificed by cervical dislocation, and the blood, lung tissue, and bronchoalveolar lavage fluid (BALF) samples were collected for analysis. The animal experiments were approved by the Institutional Animal Care and Use Committee of Jinshan Hospital, Fudan University and complied with the Guide for the Care and Use of Laboratory Animals, 8th edition.

### 2.7. Measurement of Lung Wet-to-Dry Ratio

The lung tissue samples from all groups were obtained and measured, which was subsequently used as the wet weight. The samples were placed in an incubator at 55°C overnight and their dry weight was measured subsequently. The lung wet-to-dry ratio was calculated by dividing wet weight by dry weight.

### 2.8. Measurement of Protein Concentration and Cytokine Levels in the BALF and Serum

The protein concentrations at 6, 24, and 48 h and the expression of the receptor for advanced glycation end-products (RAGE) in BALF samples were measured using a BCA protein assay kit (Beyotime) and a rat RAGE ELISA kit (Abcam, Cambridge, UK), following the manufacturer's protocols. Additionally, the levels of tumour necrosis factor-*α* (TNF-*α*), interleukin-1*β* (IL-1*β*) and interleukin-6 (IL-6) in BALF and serum samples of the groups at 6, 24, and 48 h were detected using corresponding ELISA kit (Beyotime), following manufacturer's instructions.

### 2.9. Histopathology Analysis

The lung tissues were washed and fixed with 10% formaldehyde for 24 h. The lung tissue samples were decalcified using 10% Ethylene Diamine Tetraacetic Acid solution and then embedded in paraffin. Following this, 4 *μ*m sections were cut and stained with HE. The slices were scanned and photographed using optical microscopy.

### 2.10. Western Blot

The lung tissues were used to determine the protein expression of matrix metallopeptidase 9 (MMP9) and surfactant protein C (SPC) using western blot. Briefly, the lung tissues were lysed using RIPA lysis buffer, and the protein concentrations were detected using a BCA protein assay kit. After that, the protein samples (20 *μ*g) were separated using 12% SDS-PAGE, and transferred to PVDF membranes. After sealed with 5% skimmed milk, the membranes were incubated with the primary antibodies at 4°C overnight, and then incubated with the secondary antibody at 37°C for 2 h. The primary antibodies were anti-MMP9 antibody (1 : 1000), anti-SPC antibody (1 : 1000), and anti-GAPDH antibody (1 : 3000), as well as the secondary antibody was goat anti-rabbit IgG-HRP (1 : 5000). All antibodies were purchased from Abmart Biotechnology Co., Ltd. Shanghai, China. After washing, the protein bands were visualized using an enhanced chemiluminescence (ECL) reagent, and quantified using the ImageJ software.

### 2.11. Tracking In Vivo

To observe the distribution of MSCs *in vivo*, MSCs were transfected with a lentiviral vector containing both green-fluroscence protein and luciferase reporter genes (GFP-Luc, GenePharma, Shanghai, China) based on the manufacturer's protocols. After 48 h of transfection, a fluorescent microscope (Olympus, Tokyo, Japan) was used to examine the GFP expression. The stable transfected GFP-Luc MSCs were screened using a medium containing 3 *μ*g/mL puromycin (Thermo Fisher Scientific) for 2 weeks. The luciferase activity of MSCs was evaluated using bioluminescence imaging (BLI) via an AniView 100 Living Imaging system (Guangzhou Biolight Biotechnology Co., Ltd. Shanghai, China) after 15 min of D-luciferin addition.

Additionally, the stable transfected GFP-Luc MSCs were used for tracking *in vivo*. A total of 18 SD rats were randomly distributed into three groups as follows (*n* = 6 per group): CON, MSC, and RGD-MSC groups. After phosgene exposure, the rats in the CON, MSC, and RGD-MSC groups were administered 50 *μ*L PBS, 1 × 10^6^ GFP-Luc MSCs per 50 *μ*L PBS and 1 × 10^6^ GFP-Luc MSCs per 50 *μ*L RGD-hydrogel via intratracheal drip, respectively. After 6, 24, and 48 h of treatment, the BLI signals of the obtained lung tissues were evaluated at different time points after D-luciferin intraperitoneal injection (iView 100 Living Imaging system).

### 2.12. Statistical Analyses

Statistical analyses were conducted using SPSS version 19.0 statistical software and GraphPad Prism 5.0 software. Student's *t*-test and one-way ANOVA were used for comparison between groups. The data are expressed as mean ± standard deviation, and statistical significance was set at *P* < 0.05.

## 3. Results

### 3.1. Mild Cytotoxicity of RGD-Hydrogel in MSCs

**Figure 1:**
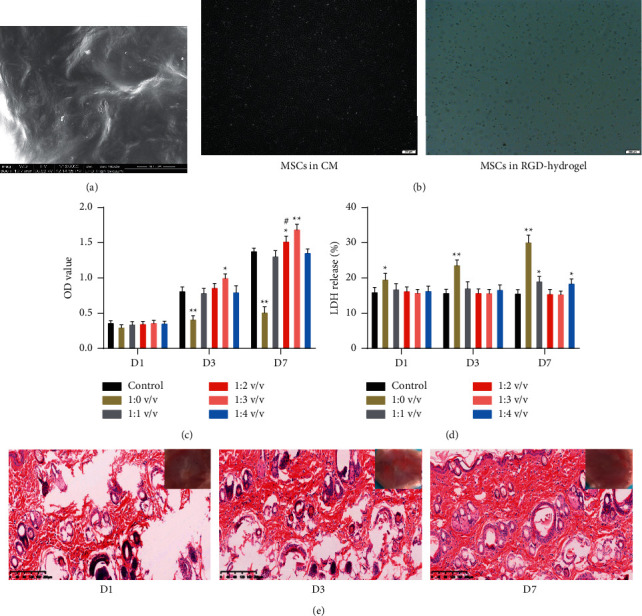
The cytotoxicity of RGD-hydrogel in bone marrow-derived MSCs. (a) SEM image of RGD-hydrogel surface (Bar = 50 *μ*m). (b) The morphology of MSCs cultured in conventional medium and RGD-hydrogel medium under a light microscope (Bar = 200 *μ*m). (c) The viability of MSCs after culturing with different concentrations of RGD-hydrogel (1 : 0, 1 : 1, 1 : 2, 1 : 3, and 1 : 4 v/v) for 1 day, 3 days, and 7 days using CCK-8 assay kit. (d) The LDH release of MSCs after culturing with different concentrations of RGD-hydrogel (1 : 0, 1 : 1, 1 : 2, 1 : 3, and 1 : 4 v/v) for 1 day, 3 days, and 7 days using LDH assay kit. (e) The biocompatibility and degradability of RGD-hydrogel in vivo after injection with 50 *μ*L RGD-hydrogel for 1 day, 3 days, and 7 days (Bar = 200 *μ*m). *∗*: *P* < 0.05, *∗∗*: *P* < 0.01, compared with the control group. #: *P* < 0.05, ##: *P* < 0.01, compared with the 1 : 3 v/v group.

RGD-hydrogel surface displayed an irregular wavelike structure (Figure 1(a)). The morphology of MSCs in conventional culture medium and medium with RGD-hydrogel was observed under a light microscope. MSCs showed a typical spindle shape in the conventional culture medium while those in the RGD-hydrogel medium showed spheroid clusters along with the adhesion of RGD-hydrogel and MSCs (Figure 1(b)). The effects of different concentrations of RGD-hydrogel on the viability of MSCs at different time points were investigated. After 1 day of treatment, no significant difference was observed in the cell viability of MSCs among the control and different RGD-hydrogel concentration groups (*P* > 0.05, Figure 1(c)). After 3 and 7 days of treatment, the viability of MSCs in the 1 : 0 v/v and 1 : 3 v/v groups significantly decreased (*P* < 0.05) and increased (*P* < 0.05), respectively; however, there was no significant difference in the viability of MSCs among the control, 1 : 1, and 1 : 4 v/v groups (*P* > 0.05, Figure 1(c)).

RGD-hydrogel-induced cytotoxicity was assessed using the LDH assay. After 1 and 3 days of treatment, LDH content was significantly higher in the 1 : 0 v/v group than that in the control group (*P* < 0.05), whereas no significant difference was observed among the control, 1 : 1, 1 : 2, 1 : 3, and 1 : 4 v/v groups (*P* > 0.05, Figure 1(d)). Additionally, after 7 days of treatment, LDH content in the 1 : 0, 1 : 1 and 1 : 4 v/v groups was significantly increased compared with the control group (*P* < 0.05); however, there was no significant difference among the control, 1 : 2, and 1 : 3 v/v groups (*P* > 0.05, Figure 1(d)). Finally, the biocompatibility and biodegradability of RGD-hydrogel were explored via the subcutaneous injection of 50 *μ*L RGD-hydrogel on the back of rats. Obvious tissue injury was observed on day 1 after injection, and the RGD-hydrogel was partially absorbed on day 3 and completely absorbed on day 7 (Figure 1(e)). Therefore, RGD-hydrogel had little cytotoxicity, revealing its biocompatible and biodegradable *in vivo*.

### 3.2. Effects of RGD-Hydrogel on MSC-Secreted Cytokines

**Figure 2:**
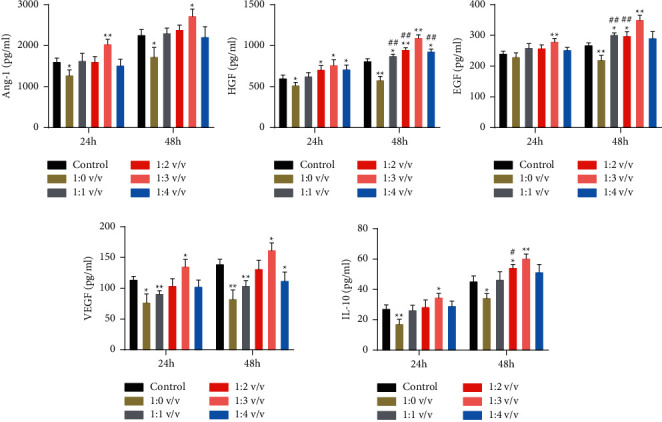
Effects of different RGD-hydrogel concentrations on MSC-secreted cytokine levels (Ang-1, HGF, EGF, VEGF, and IL-10) at 24 h and 48 h using corresponding ELISA kits. *∗*: *P* < 0.05, *∗∗*: *P* < 0.01, compared with the control group. #: *P* < 0.05, ##: *P* < 0.01, compared with the 1 : 3 v/v group.

MSC-secreted cytokine levels were determined in the MSCs cultured in different concentrations of RGD-hydrogel and conventional culture medium at 24 and 48 h. After culturing for 24 and 48 h, the levels of Ang-1 and IL-10 were significantly reduced in the 1 : 0 v/v RGD-hydrogel medium compared with the conventional medium (*P* < 0.05), whereas it was significantly increased in the 1 : 3 v/v RGD-hydrogel medium (*P* < 0.05, Figure 2). For HGF, EGF, and VEGF, after treatment for 24 and 48 h, their levels were significantly decreased in the 1 : 0 v/v RGD-hydrogel medium compared with that of the control (*P* < 0.05). Moreover, their levels gradually increased with increasing RGD-hydrogel concentration, peaking and plunging in the 1 : 3 v/v and 1 : 4 v/v RGD-hydrogel mediums, respectively (Figure 2). The 1 : 3 v/v RGD-hydrogel concentration was chosen for subsequent experiments owing to its non-cytotoxicity and significant MSC-secreted cytokine levels.

### 3.3. Effects of RGD-Hydrogel-Encapsulated MSCs on the P-ALI Rat Model

**Figure 3:**
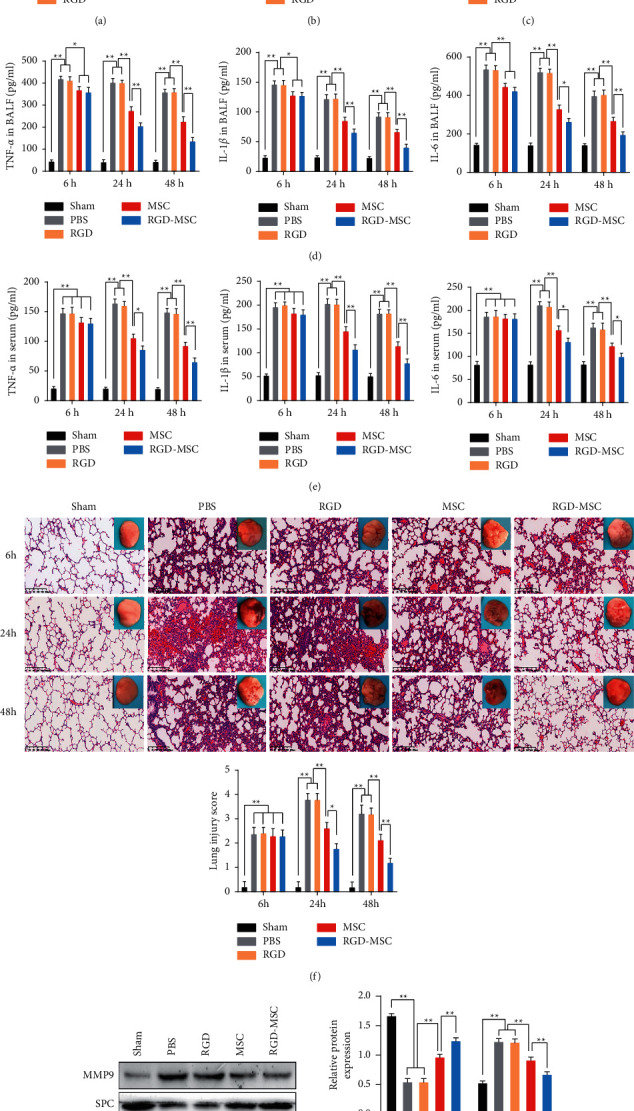
Effects of the hydrogel-encapsulated MSCs on P-ALI rat model. (a) The lung wet-to-dry ratio in the groups after 6, 24, and 48 h of interference. (b) Total protein concentration in BALF samples of the groups at different time points. (c) The levels of receptor expression for RAGE in BALF samples of the groups after 6, 24, and 48 h of treatment. (d) The levels of TNF-*α*, IL-1*β* and IL-6 in BALF samples of different groups after 6, 24, and 48 h of treatment using ELISA kits. (e) The levels of TNF-*α*, IL-1*β* and IL-6 in serum samples of different groups after 6, 24, and 48 h of treatment using ELISA kits. (f) Histological changes in lung tissues of the groups using HE staining (Bar = 200 *μ*m). (g) The protein expression of SPC and MMP9 in lung tissues of different groups by western blot. *∗*: *P* < 0.05, *∗∗*: *P* < 0.01.

After 6 h of interference, the wet-to-dry ratio of lung tissues and total protein in BALF samples were significantly elevated in rats exposed to phosgene (*P* < 0.05), whereas MSCs and hydrogel-encapsulated MSCs showed no significant difference (*P* > 0.05, Figures 3(a) and 3(b)). On treatment for 24 h or 48 h, the wet-to-dry ratio and total protein in BALF samples showed a significant increase in the P-ALI rats (*P* < 0.05); however, no significant difference was found between the PBS and RGD groups (*P* > 0.05, Figures 3(a) and 3(b)). Compared to the PBS group, MSCs and hydrogel-encapsulated MSCs showed a significant reduction in the wet-to-dry ratio and total protein in BALF samples (*P* < 0.05). Moreover, the roles of hydrogel-encapsulated MSCs were more significant than the MSC group (*P* < 0.05, Figures 3(a) and 3(b)). Furthermore, the levels of RAGE in the BALF samples of the groups at different time points were measured. The RAGE levels in all BALF samples at different time points were similar to that of the total protein levels (Figure 3(c)). TNF-*α*, IL-1*β* and IL-6 levels in BALF samples were increased after phosgene exposure compared to that of the Sham group (*P* < 0.05), and there was no significant difference between the PBS and RGD groups (*P* > 0.05, Figure 3(d)). However, after the P-ALI rats were treated with MSCs and hydrogel-encapsulated MSCs, BALF levels were significantly decreased (*P* < 0.05) while no significant difference was found between the MSC and RGD-MSC groups at 6 h of interference (*P* > 0.05); however, at 24 h or 48 h, the effects of hydrogel-encapsulated MSCs were more significant than the MSC group (*P* < 0.05, Figure 3(d)). TNF-*α*, IL-1*β* and IL-6 levels in the serum showed a similar trend to the RAGE level in the BALF samples (Figure 3(e)).

The representative images of HE staining in different groups are shown in Figure 3(f). The evaluation of the lung injury scores in the groups at different time points showed normal lung tissue in the Sham group but extensive lesions in the phosgene-exposed group, including alveolar oedema and inflammatory cell accumulation, significantly damaged alveolar epithelial cells and alleviated lesions in the MSCs and hydrogel-encapsulated MSCs (Figure 3(f)). On analysing the lung injury scores, the PBS group showed a significant increase compared with that of the Sham group (*P* < 0.05). However, after 24 h and 48 h of treatment, the score was significantly reduced in the MSCs and hydrogel-encapsulated MSCs compared to the P-ALI rats treated with PBS and RGD-hydrogel (*P* < 0.05). Notably, the effects of hydrogel-encapsulated MSCs were significant (*P* < 0.05, Figure 3(f)). Finally, a western blot was employed to detect the protein expression levels of MMP9 and SPC at 24 h of administration. As shown in Figure 3(g), phosgene exposure significantly downregulated SPC protein (*P* < 0.01), while notably up-regulated MMP9 protein (*P* < 0.01) compared with the Sham group. There were no significant differences in the SPC and MMP9 expression between the PBS and RGD groups (*P* > 0.05, Figure 3(g)). However, MSCs and hydrogel-encapsulated MSCs remarkably reversed the expression of SPC and MMP9 induced by phosgene, and the effects of hydrogel-encapsulated MSCs were better (*P* < 0.05, Figure 3(g)). All these results implied that hydrogel-encapsulated MSCs could have a better therapeutic effect on P-ALI than MSCs.

### 3.4. Tracking of MSCs and RGD-Hydrogel-Encapsulated MSCs in Lung

**Figure 4:**
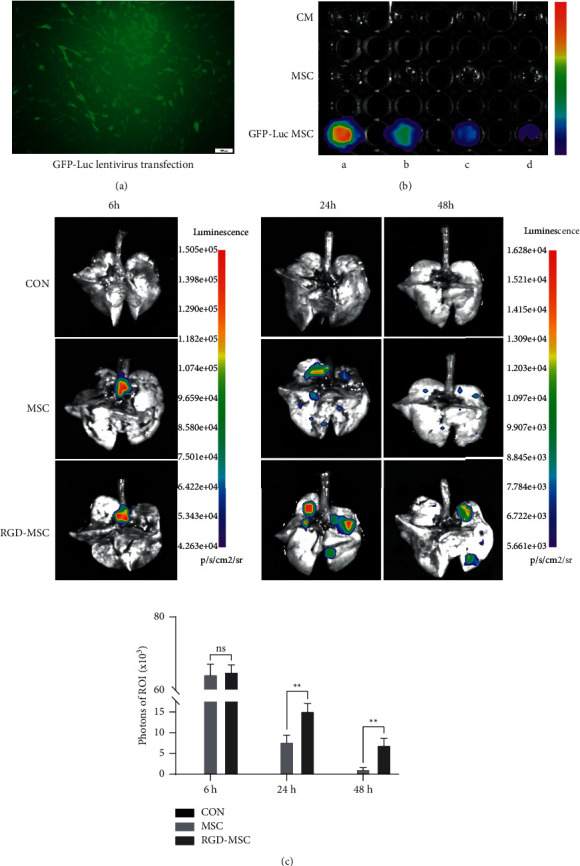
Tracking of MSCs and hydrogel-encapsulated MSCs in lung tissues. (a) The fluorescence of MSCs transfected with a lentiviral vector containing GFP-Luc (Bar = 100 *μ*m). (b) The BLI signals of GFP-Luc-MSCs with concentration gradients. (c) The BLI signals of GFP-Luc-MSCs and GFP-Luc-hydrogel-encapsulated MSCs in lung tissues after 6, 24, and 48 h of treatment. *∗*: *P* < 0.05, *∗∗*: *P* < 0.01.

MSCs were transfected with a lentiviral vector containing both GFP and luciferase reporter genes (GFP-Luc), and green fluorescence was found in MSCs after transfection (Figure 4(a)). The transfected MSCs were gradually diluted at 50%, which showed that BLI signals were consistent with the concentration gradients (Figure 4(b)). The stable transfected MSCs and hydrogel-encapsulated MSCs were injected into rats to observe the BLI signals in lung tissues at 6, 24, and 48 h. Colour-coded imaging showed that the BLI signals were significantly stronger after hydrogel-encapsulated MSCs treatment for 24 h and 48 h compared with that of MSCs (*P* < 0.01); however, no significant difference in the BLI signals was observed after 6 h of treatment (*P* > 0.05, Figure 4(c)).

## 4. Discussion

Phosgene exposure leads to inflammatory infiltration, oedema, and epithelial necrosis in the lung [22, 23]. MSCs have been reported to reduce inflammation severity and regulate endothelial permeability by releasing anti-inflammatory cytokines and growth factors [24]. However, MSCs entering the injured lung via intravenous or intratracheal infusion initially suffer severe cell death due to the pulmonary first-pass effect and harsh microenvironment, minimising the therapeutic effects [25]. The success of MSCs therapies depends heavily on the cell survival, engraftment, and the appropriate delivery systems that can match desired therapeutic applications [16]. This study uses a polysaccharide hydrogel modified with integrin-RGD peptide to mimic the natural ECM, observing its effect on the ability of bone marrow-derived MSC therapeutics in P-ALI. To our knowledge, this study is the first to combine MSCs and biomaterial RGD-hydrogel for the treatment of respiratory diseases.

The RGD peptide creates cell-adhesive structures in the hydrogel and greatly improves the long-term cell viability in three-dimensional cultures [26]. To evaluate the effects of RGD-hydrogel on bone marrow-derived MSC activities, the cells were cultured with RGD-hydrogel diluted at different concentrations. As shown in Figures 1(c) and 1(d), the 1 : 3 v/v RGD-hydrogel concentration was found to be optimal for cell survival and proliferation *in vitro*. Wang et al. found that the most suitable concentration for nucleus pulposus derived MSCs was 1 : 2 v/v [27]. Moreover, RGD-hydrogel without dilution facilitated *β*-cell proliferation [28]. This study's deviation from previous reports could be attributed to the fact that different cells require different levels of crosslinking hydrogel to match optimal nutrient transport and cell scaffold. Moreover, the current study indirectly evaluates the degradation of RGD-hydrogel *in vivo*. As shown in Figure 1(e), the tissue damage at the subcutaneous injection site largely disappeared after 7 days, indicating the good biocompatibility of RGD-hydrogel. Similarly, RGD-hydrogel was reported to gradually diminish after subcutaneous injection, with the generation of blood capillaries that could pass through the hydrogel without lymphocyte infiltration [28]. Additionally, MSC-secreted cytokine levels in the current study were increased in the 1 : 3 v/v RGD-hydrogel concentration (Figure 2), which coincides with the optimal cell proliferation results at this dilution. Ogle et al. found that MSCs cultured on RGD-hydrogel broadly enhanced immunomodulatory and regenerative factor secretions and significantly reduced cell senescence compared with those cultured on plastic surfaces [29]. Additionally, the gene expression of HGF and IL-10 in MSCs was significantly upregulated by the RGD-hydrogel culture system *in vitro* [17]. Therefore, the 1 : 3 v/v RGD-hydrogel concentration was selected for subsequent *in vivo* experiments.

The potential of RGD-hydrogel as an MSC delivery vehicle was evaluated by encapsulating the MSCs with RGD-hydrogel and administering them to rats with P-ALI. The level of inflammation, pathological changes, and tissue edema were used to assess the severity of lung injury. As shown in Figure 3, both MSCs and MSCs combined with RGD-hydrogel promoted the healing of P-ALI by inhibiting inflammatory cytokines and the ALI marker RAGE, reducing pulmonary oedema and inflammatory infiltration, increasing SPC expression and suppressing MMP9 expression. Moreover, from these test results, MSCs combined with RGD-hydrogel showed better therapeutic effects than MSCs alone. An analogical study also showed that the epicardial placement of MSC-loaded hydrogel promoted the recovery of cardiac function and structure with reduced interstitial fibrosis in a rat myocardial infarction model [30]. Although the relevant signalling pathway mechanism are yet to be explored, the upregulation of five MSC-secreted therapeutic factors (Figure 2) allowed the speculation that the hydrogel-encapsulated MSCs ameliorate P-ALI by enhancing cell paracrine functions.

The bioluminescent imaging signal was used to estimate the number of exogenous MSCs in the lung. Cell tracking showed that the number of both MSCs and hydrogel-encapsulated MSCs reduced within 48 hours after endotracheal drip (Figure 4(c)), which could be attributed to the fact that engraftment in the injured lung does not occur easily [31, 32]. Nevertheless, the hydrogel-encapsulated MSCs were more retained after the intervention (Figure 4(c)), indicating that RGD-hydrogel as a delivery vehicle could improve MSC survival in the injured lung. RGD-hydrogel improved MSC survival but not cell engraftment in the lung. This result varied with the orthotopic transplantation of exogenous MSCs into dense tissues, such as ischemic myocardium [33] and injured brain [34], which could be attributed to the insufficient adhesion between the cells and lung lacunar parenchyma [35]. Hence, RGD-hydrogel has the potential to enhance lung injury repair by increasing MSC secretion and survival. Despite *in vitro* and *in vivo* results, the mechanisms of RGD-hydrogel enhancing the therapeutic effect of MSCs in P-ALI remain unclear and require further study.

## 5. Conclusion

RGD-hydrogel could promote the paracrine function of MSCs *in vitro* and cell survival *in vivo*. MSCs combined with RGD-hydrogel further alleviate P-ALI via intratracheal infusion. This study provides a foundation for the application of hydrogel in exogenous MSC treatment of ALI.

## Data Availability

The data used in this work are available from the corresponding author on reasonable request.
